# The *hetero*-Friedel-Crafts-Bradsher Cyclizations with Formation of Ring Carbon-Heteroatom (P, S) Bonds, Leading to Organic Functional Materials

**DOI:** 10.3390/ma13214751

**Published:** 2020-10-23

**Authors:** Joanna Skalik, Marek Koprowski, Ewa Różycka-Sokołowska, Piotr Bałczewski

**Affiliations:** 1 Division of Organic Chemistry, Centre of Molecular and Macromolecular Studies, Polish Academy of Sciences, Sienkiewicza 112, 90-363 Łódź, Poland; mkopr@cbmm.lodz.pl; 2 Institute of Chemistry, Faculty of Science and Technology, Jan Długosz University in Częstochowa, Armii Krajowej 13/15, 42-201 Częstochowa, Poland; ewacrystal@gmail.com

**Keywords:** sulfur, phosphorus, sulfoxide, *thia*-Friedel-Crafts-Bradsher, *phospha*-Friedel-Crafts-Bradsher, cyclization, heteroacene, thiophene, phosphole, application

## Abstract

The interest in functional materials possessing improved properties led to development of new methods of their synthesis, which allowed to obtain new molecular arrangements with carbon and heteroatom motifs. Two of the classical reactions of versatile use are the Friedel-Crafts and the Bradsher reactions, which in the new heteroatomic versions allow to replace ring carbon atoms by heteroatoms. In the present work, we review methods of synthesis of C–S and C–P bonds utilizing *thia*- and *phospha*-Friedel-Crafts-Bradsher cyclizations. Single examples of C–As and lack of C–Se bond formation, involving two of the closest neighbors of P and S in the periodic table, have also been noted. Applications of the obtained π-conjugated molecules, mainly as semiconducting materials, flame retardants, and resins hardeners, designed on the basis of five- and six-membered cyclic molecules containing ring phosphorus and sulfur atoms, are also included. This comprehensive review covers literature up to August 2020.

## 1. Introduction

During the last years, π-conjugated molecules containing various ring heteroatoms, such as boron, nitrogen, silicon, sulfur, oxygen, or phosphorus atoms incorporated into polycyclic ring systems gained an increasing importance due to their unique optical and electronic properties which made them useful functional materials, especially in the area of semiconducting materials [1]. Two elements of the fifth and sixth groups of the periodic table, sulfur and phosphorus, occupy a special position in the heteroatom family. Despite the close proximity in main groups and in the third row of the table, the properties of materials containing these two heteroatoms vary considerably due to differences in atomic radii, electronegativities, and a number of free electron pairs available. They are incorporated in five- or six-membered ring systems, mainly thiophene and phosphole derivatives. The latter possesses lower resonance energy compared to the former, originating from differences in configuration of phosphorus (flattened sp^3^) and sulfur (sp^2^) atoms. Thus, phosphole has nearly six times weaker aromatic character than thiophene based on aromatic stabilization energies (ASE_calc_ = 3.2 versus 18.57 kcal mol^−1^) [2]. These differences, however, may be an asset, utilized in modulating the material properties based on these molecules. Both phosphorus and sulfur can be present in pendant hetero-organic groups outside the ring [3,4] or can replace one of the ring carbon atoms of the molecular backbones. The latter situation concerns the subject matter of this comprehensive review, which covers literature up to August 2020.

**Scheme 1:**
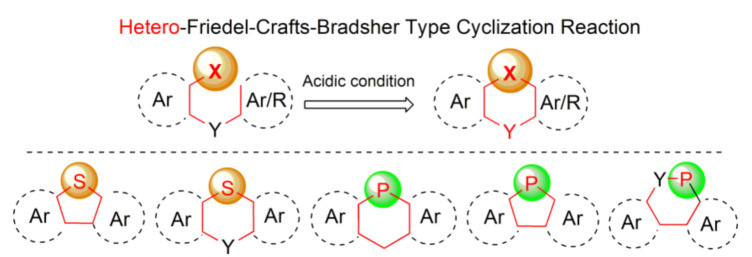
The *thia*- and *phospha*-Friedel-Crafts-Bradsher reactions outlined in this review.

Among various general methods for the synthesis of arenes and heteroarenes, the Bradsher cyclization (1940), which is the intramolecular version of the discovered earlier Friedel-Crafts acylation and alkylation reactions (in 1877), realizes the most common ring closure strategy. From the mechanistic point of view, this reaction is categorized as the electrophilic aromatic substitution S_E_Ar, in which a carbocation σ complex (arenium ion) is formed as an intermediate. Nearly all organic compounds of electrophilic character may react with (hetero)aromatics in this reaction under suitable activation conditions. It is also known that heteroatom cations generated from p-block-elements including phosphorus and sulfur may also interact with (hetero)arenes to form heteroarenium species and thus enable introduction of ring heteroatoms in place of carbon atoms. This type of modification, which may be referred as a *hetero*-Friedel-Crafts-Bradsher reaction, is a useful tool for incorporation of heteroatoms into π-conjugated frameworks. Our intention was to summarize, in this review, all synthetic approaches based on mechanistic principles of this reaction leading to five- and six-membered cyclic products via the heteroatom–carbon bond formation. An integral part of this work is a review of properties and applications of the synthesized molecules, which is placed in the end of each subsection. Lack of information means that only synthesis of a new molecule or a group of molecules has been developed. Most of the data, especially in case of thiophene-based materials, concerns opto-electric properties due to the unique structures of the obtained molecules containing considerable unsaturation in addition to phosphorus and sulfur, accompanied by other ring heteroatoms, like oxygen and nitrogen (Scheme 1). Some selected examples of the *hetero*-Friedel-Crafts-Bradsher cyclization may be found in earlier literature [1,5].

## 2. Cyclisation of Sulfur Derivatives

The first literature report concerning intermolecular Friedel-Crafts sulfinylation of aromatic compounds, starting from diaryl or alkyl aryl sulfoxides, was published in 1926 by Farah and coworkers [6]. After 30 years, Douglas et al. reported the synthesis of methyl phenyl sulfoxide from benzene and methanesulfinyl chloride in the presence of AlCl_3_ [7]. Also, Gupta published the synthesis of aromatic *N*,*N*-dialkylsulfonamides via the reaction of dialkylsulfamyl chlorides with aromatic hydrocarbons in the presence of AlCl_3_ [8]. On the other hand, Yuste and coworkers reported that the intermolecular Friedel-Crafts reaction of alkyl- and arylsulfinates with aromatic systems, activated by electron-donating substituents, provided alkyl aryl and diaryl sulfoxides under mild conditions in moderate to good yields [9]. 

**Scheme 2:**
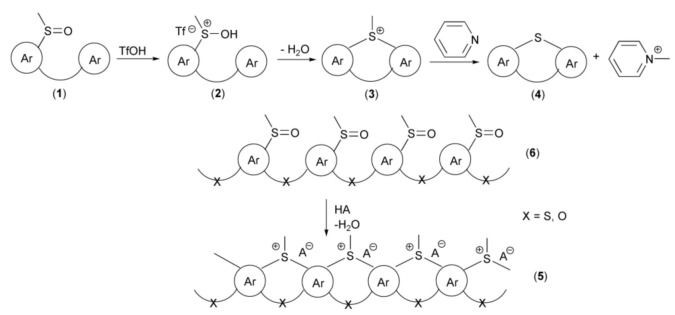
The reaction mechanism of the *thia*-Friedel-Crafts-Bradsher annulation leading to the synthesis of polycyclic systems containing a ring sulfur atom.

One of the most efficient key approaches for the synthesis of polycyclic systems containing ring sulfur atom and utilizing intramolecular electrophilic annulation reaction of sulfoxide groups in alkylsulfinylarenes with aromatic rings was introduced by Müllen and coworkers in 1999. The mechanism of this transformation assumed protonation of oxygen atom in the sulfoxide group (**1**), in the presence of trifluoromethanesulfonic acid (triflic acid, TfOH) with formation of electrophilic sulfonium cation (**2**). Thus, this acidic activation of sulfoxide resembles activation of carbonyl group in the presence of Brönsted or Lewis acids in S_E_Ar reactions. In the next step, the sulfonium cation (**2**) underwent intramolecular, electrophilic substitution reactions followed by elimination of water, to give cyclic methylsulfonium salt (**3**). In the last step, a base, usually pyridine, promoted demethylation to provide the derivative (**4**). The above strategy was utilized in syntheses of not only “small molecules” but also polymeric systems (**5**) starting from polysulfoxides (**6**) as substrates (Scheme 2).

**Scheme 3:**
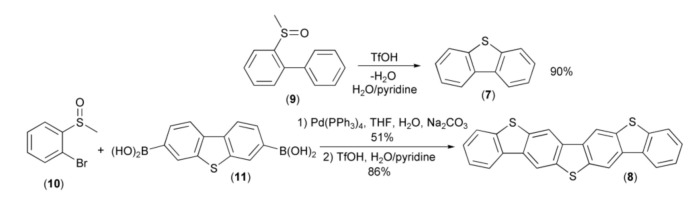
The synthesis of dibenzothiophene (**7**) and dibenzo[*b,b’*]thieno[2,3-f:5,4-*f’*]bis[1] benzothiophene (**8**).

The first illustration of this new methodology was synthesis of dibenzothiophene (**7**) and dibenzo[*b*,*b*’]thieno[2,3-*f*:5,4-*f*’]bis[1]benzothiophene (DBTBT, **8**) reported by Sirringhaus and coworkers. The syntheses proceeded via the acid-induced intramolecular cyclization reaction of arenes containing methyl sulfoxide groups, i.e., methylsulfinylbiphenyl (**9**) and 3,7-bis(methylsulfinylophenyl)dibenzothiophene. The latter was prepared through the Suzuki–Miyaura cross-coupling reaction of the bromide (**10**) and the diboronic acid (**11**) (Scheme 3) [10].

**Scheme 4:**
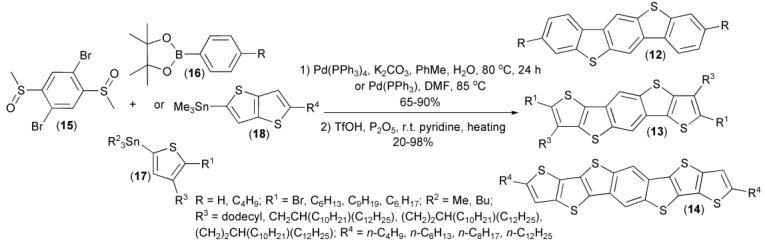
The synthesis of substituted and unsubstituted benzothiophene derivatives (**12**–**14**).

A similar approach was utilized by Müllen and coworkers in a two-step synthesis of substituted and unsubstituted benzo[1,2-*b*:4,5-*b*’]bis[*b*]benzothiophenes (**12**), dithieno[2,3-*d*;2′,3′-*d*’]benzo[1,2-*b*:4,5-*b*’]dithiophenes (**13**), and dithienothieno[2,3-*d:*2′,3′-*d’*]benzo[1,2-*b*:4,5-*b’*]dithiophenes (**14**), as useful materials for organic transistors. In this synthesis, 2,5-dibromophenyl dimethyl disulfoxide (**15**), a cyclic boronic ester (**16**), or organotin compounds (**17**, **18**) were applied as the corresponding substrates (Scheme 4) [11,12,13,14,15,16].

**Scheme 5:**
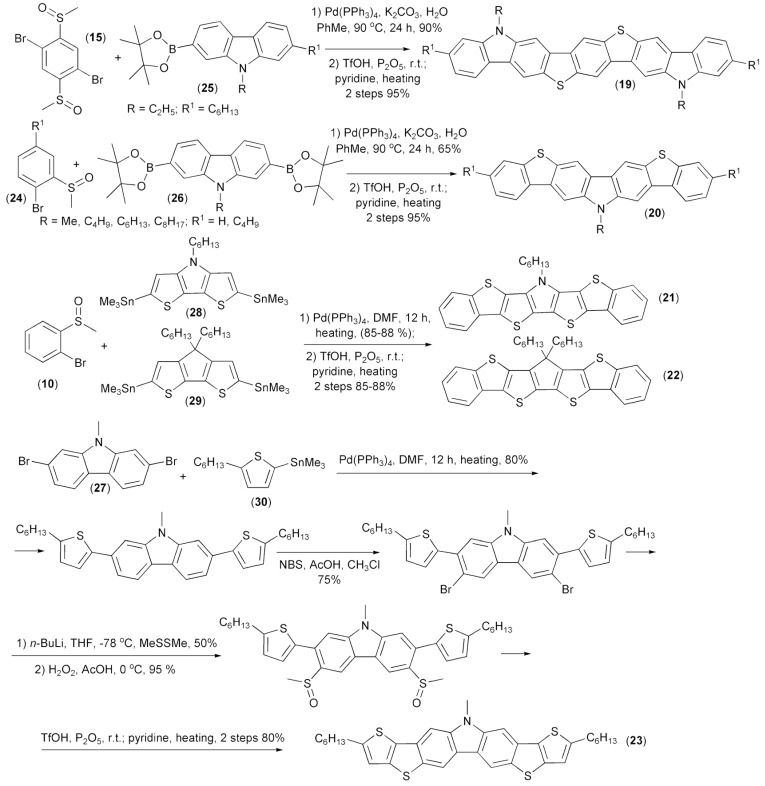
The synthesis of heteroacene derivatives containing carbazole, pyrrole, or thiophene aromatic rings (**19**–**23**).

The same research group synthesized heteroacenes (**19–23**) containing carbazole, pyrrole, or thiophene aromatic rings. A key step of these syntheses involved intramolecular triflic acid (TfOH)-mediated cyclization. The corresponding substrates for the cyclizations were prepared through the Suzuki cross-coupling reaction of 2,5-dibromophenyl dimethyl sulfoxide (**15**) or 1-bromophenyl methyl sulfoxides (**24**) with carbazole 2-boronic (**25**) or 2,7-diboronic acids diesters (**26**) and the Stille coupling reaction of 1-bromophenyl methyl sulfoxides (**24**) or 2,7-dibromocarbazole (**27**) with ditin heteroaryl derivatives (**28**, **29**) or tin heteroaryl derivative (**30**) (Scheme 5) [17,18].

**Scheme 6:**
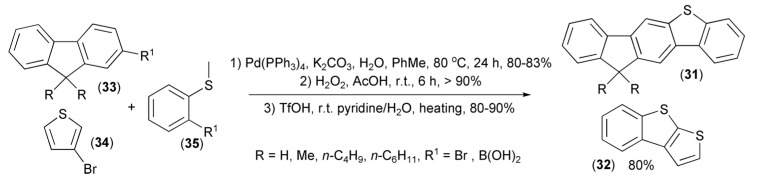
The preparation of fluoreno[2,3-*b*]benzo[*d*]thiophene (**31**) and benzothieno[2,3-*b*]thiophene (**32**).

Application of a similar strategy allowed preparation of unsymmetrical fluoreno[2,3-*b*]benzo[*d*]thiophene (**31**) and benzothieno[2,3-*b*]thiophene (**32**) from fluorenes (**33**), 3-bromothiophene (**34**), and sulfides (**35**) (Scheme 6). Photophysical properties showed that these compounds can be considered as effective dopant materials for organic emitting diodes [19,20].

**Scheme 7:**
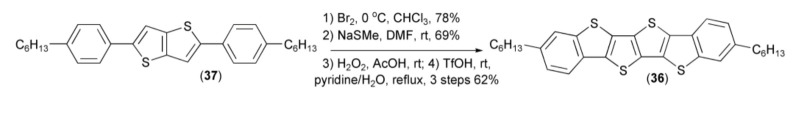
The preparation of dibenzotetrathienoacene (**36**).

Liu and coworkers prepared dibenzotetrathienoacene (**36**) through the following reaction sequence: bromination of the teraryl (**37**), reaction of the resulting bromo-derivative with NaSMe, oxidation with hydrogen peroxide, followed by the final cyclization using triflic acid (Scheme 7) [21].

**Scheme 8:**
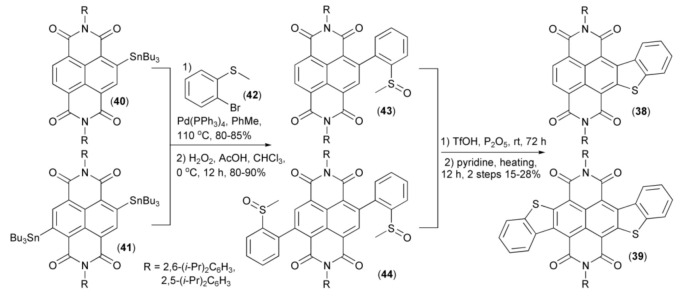
The preparation of naphtalenodiimides (**38**) and (**39**).

Gao and coworkers employed the Stille coupling for synthesis of benzothiophene-fused naphtalenodiimides (**38**) and (**39**) from 2- and 2,6-substituted tin derivatives (**40**) and (**41**), respectively. The latter was first reacted with 2-bromothioanizole (**42**) and then oxidized with hydrogen peroxide to give sulfoxides (**43**) and (**44**), respectively, and finally cyclized to the desired products (**38**) and (**39**) in the presence of triflic acid (TfOH)/phosphorus pentoxide (Scheme 8) [22].

**Scheme 9:**
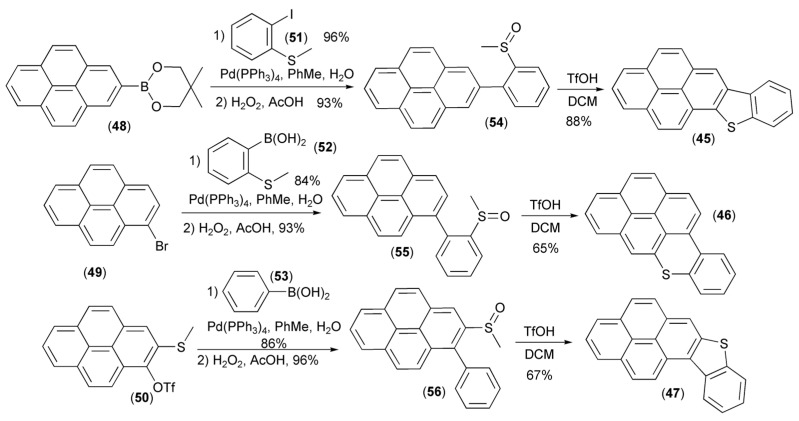
The synthesis of new pyrene fused thienoacenes (**45**–**47**).

Three approaches to the synthesis of new pyrene fused thienoacenes (**45–47**) were realized by Li and coworkers. The approaches utilized a coupling of pyrene derivatives (**48–50**) with 2-iodothioanisol (**51**) or boronic acids (**52**)/(**53**), and subsequent oxidation to afford sulfoxides (**54–56**), which in the final step underwent cyclization to regioisomeric [3,4]-, [1,2]-, and [2,1]-pyrenes (**45–47**) in the presence of triflic acid (TfOH) (Scheme 9) [23].

**Scheme 10:**
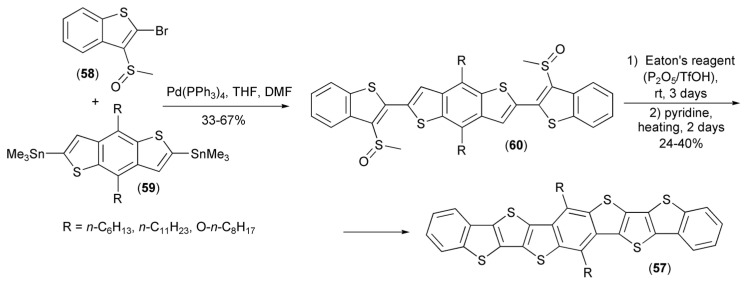
The synthesis of thieno[3,2-*b*;4,5-*b’*]-dithiophenes (**57**).

Similar synthetic methodology was used by Li and coworkers for the synthesis of thieno[3,2-*b*;4,5-*b’*]-dithiophenes (**57**). In this approach, 2-bromo-3-methylsulfinylbenzothiophene (**58**) underwent the Stille coupling reaction with tin derivatives (**59**) providing teraryls (**60**), which were next cyclized in the presence of the Eaton’s reagent (7.7 wt% P_2_O_5_ in CH_3_SO_3_H) and finally aromatized with pyridine to afford the desired products (**57**) (Scheme 10) [24]. Further investigations showed that compound (**57**) had suitable HOMO energy levels to be applied in organic transistors as p-channel organic semiconductors. Thin-film transistor characteristics showed that all derivatives displayed a high device reproducibility and nearly no dependence on substrates temperature.

**Scheme 11:**
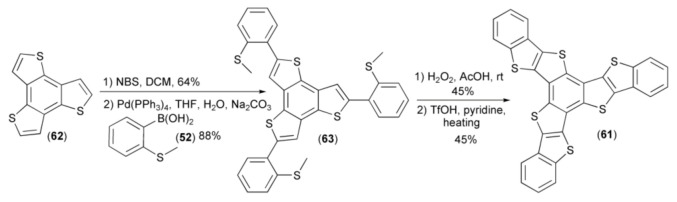
The synthesis of benzo[1,2-*b*:3,4-*b’*:5,6-*b’’*]-tri(benzo[4′,5′]thieno[2′,3′-*d’*]thiophene) (**61**).

Based on the described methodology, Hu and coworkers synthesized benzo[1,2-*b*:3,4-*b’*:5,6-*b’’*]-tri(benzo[4′,5′]thieno[2′,3′-*d’*]thiophene) (**61**) starting from symmetrical benzo[1,2-*b*:3,4-*b’*:5,6-*b’’*]-trithiophene (**62**). Bromination of (**62**) followed by coupling with boronic acid (**52**) afforded the trisulfide (**63**). Oxidation of the latter in the next step and cyclization in the presence of triflic acid (TfOH) provided polycyclic heteroacene system (**61**) in 45% yield (Scheme 11) [25].

**Scheme 12:**
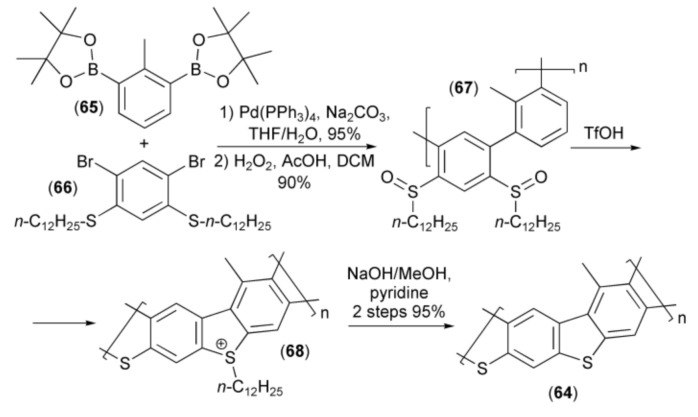
The synthesis of poly(thiaheterohelicene) (**68**).

Nishide and coworkers synthesized poly(thiaheterohelicene) (**64**) containing dibenzothiophene moieties. In this synthesis, the palladium catalyzed polycondensation of cyclic diboronic acid diesters (**65**) with 2,4-dibromo-1,5-di-*n*-dodecylthiobenzene (**66**) followed by oxidation of the sulfide to sulfoxide function with hydrogen peroxide provided polyphenylene (**67**), which was cyclized to the corresponding dibenzothiophene polymer (**64**) under strong acidic conditions via sulfonium derivative (**68**) (Scheme 12) [26].

**Scheme 13:**
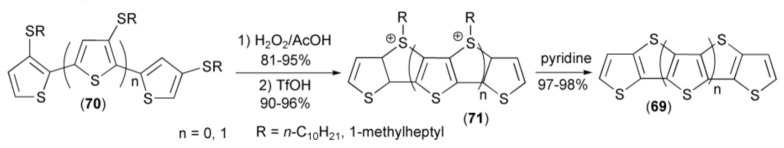
The synthesis of a new type of polythiophene oligomers (**69**).

Tuschida and coworkers synthesized a new type of polythiophene oligomers (**69**) by the cyclization reaction of bithienyls or terthienyls (**70**) to sulfonium derivatives (**71**) followed by aromatization with pyridine to give fused thieno-systems (**69**) (Scheme 13) [27].

**Scheme 14:**
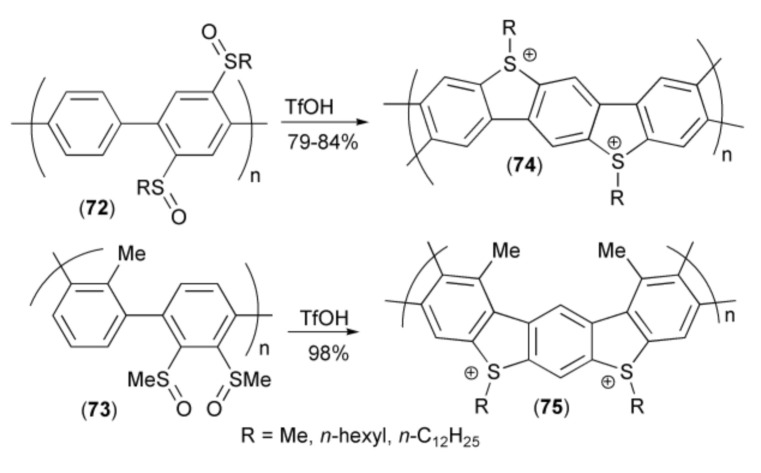
The synthesis of ionic polymers (**74**, **75**).

Intramolecular ring closing reaction of alkylsulfinyl-polypara(phenylenes) (**72**, **73**) in the presence of triflic acid (TfOH) provided polymers (**74**, **75**) possessing ionic character (Scheme 14) [28,29].

**Scheme 15:**
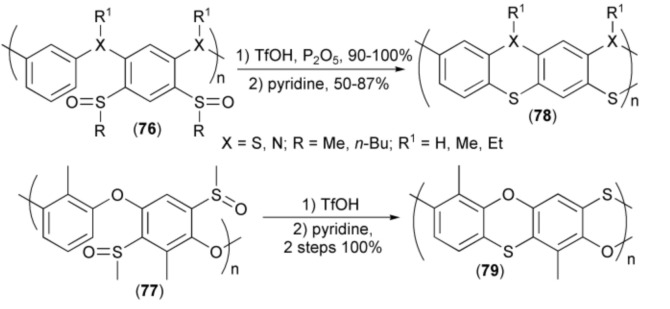
The synthesis of oligomeric and polymeric systems (**78**, **79**) containing ring sulfur in addition to ring nitrogen and oxygen atoms.

Cyclisation of sulfoxide systems was also utilized in synthesis of oligomeric and polymeric systems containing other heteroatoms (X = O, N) in addition to sulfur. Starting from precursors (**76**, **77**), the corresponding dithianhtrene (9,10-dithia-anthracene), polyphenothiazine, as well as phenoxantine systems (**78**, **79**) with imine, ether, and thioether bridges, respectively, were obtained in good to excellent yields (Scheme 15) [30,31,32,33,34,35,36].

**Scheme 16:**
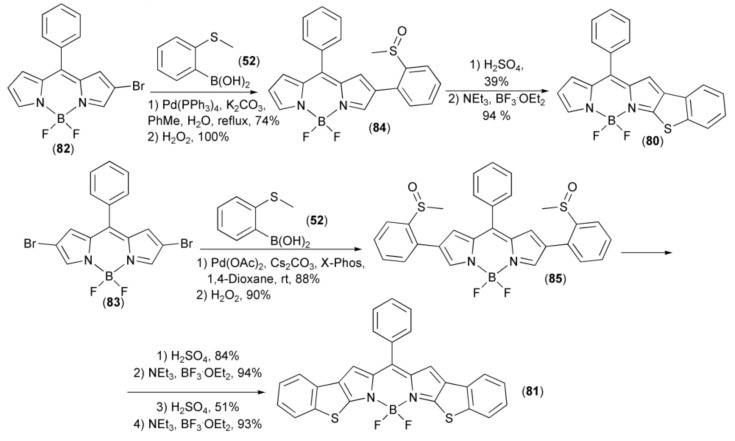
The synthesis of benzothieno[*b*]-fused BODIPYs (**80**) and (**81**).

Two benzothieno[*b*]-fused BODIPYs (**80**) and (**81**) were synthesized from mono- and dibrominated BODIPY precursors (**82**) and (**83**). In this synthesis, the Pd-catalyzed Suzuki–Miyaura cross-coupling reactions of the latter with 2-(methylthio)-phenylboronic acid (**50**) afforded, after oxidation with hydrogen peroxide, intermediate 2-(methylsulfinyl) phenyl-substituted BODIPY derivatives (**84**) and (**85**). The intramolecular cyclization of the mono-sulfoxide (**84**) with H_2_SO_4_ (neat), led to the cyclic intermediate product without boron, which then was borylated with BF_3_·OEt_2_ to give BT-BODIPY (**80**). However, the reaction of the bis-sulfoxide (**85**) with H_2_SO_4_ (neat) led to the mono-cyclization product only, in 84% yield. This product was complexed with BF_3_·OEt_2_ and again submitted to strong acidic conditions H_2_SO_4_ (neat), which made cyclization of the second sulfoxide moiety effective in 51% yield. The resulting intermediate which lost boron was complexed with BF_3_·OEt_2_ to form the desired benzothieno[*b*]-fused BODIPYs (**81**). Finally, it was found that both BT-BODIPY (**80**) and BBT-BODIPY (**81**) displayed red shifted absorption, increased molar absorptivity, and fluorescence efficiency (Scheme 16) [37].

**Scheme 17:**
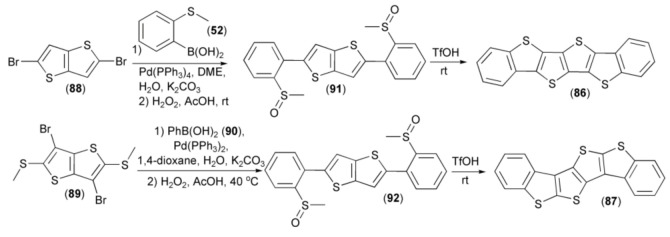
The synthesis of tetrathienoacenes (**86**, **87**).

Two tetrathienoacenes (**86**, **87**) were obtained in a moderate yield by the Suzuki cross-coupling reaction of thieno[3,2-*b*]thiophenes (**88**, **89**) with boronic acids (**50**, **90**) and subsequent oxidation of sulfur atoms in the corresponding sulfides to sulfoxides (**91**, **92**) using 30% aqueous hydrogen peroxide. Cyclization of the latter derivatives (**91**, **92**) delivered the target compounds (**86**, **87**) in 75% yield. Measurements of charge mobilities (0.15 for (**86**) and 0.047 cm^2^/V s) for (**87**)) revealed that the linear C2-symmetrical isomer (**86**) was a better candidate for fabrication of organic field-effect transistors than the kinked one (**87**) (Scheme 17) [38].

**Scheme 18:**
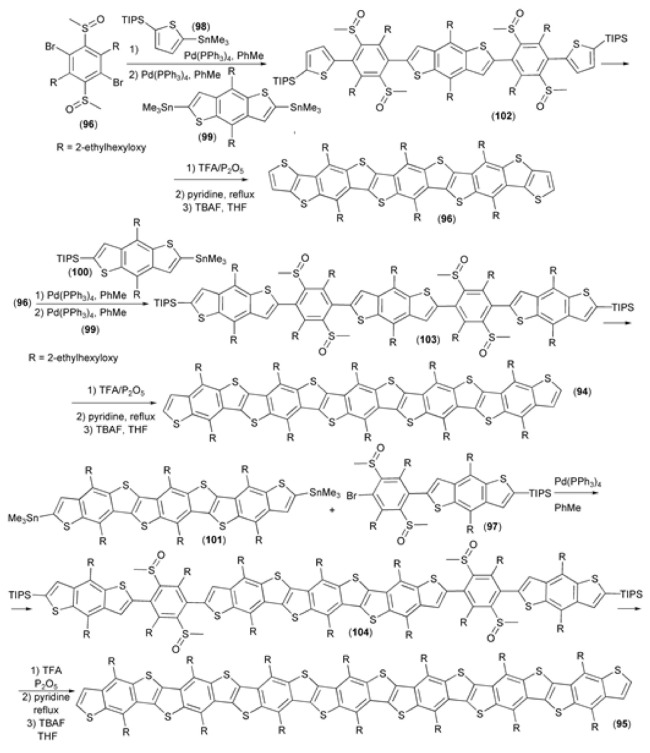
The synthesis of thienoacenes (**93**–**95**) based on the benzo[1,2-*b*:4,5-*b′*]dithiophene skeleton.

A series of soluble ladder-type thienoacenes (**93**–**95**) based on benzo[1,2-*b*:4,5-*b′*]dithiophene (BDT) skeleton were synthesized and characterized as p-type semiconductors with wide band gaps. The synthetic strategy, which delivered derivatives (**93**–**95**), included two key steps: the cross-coupling of bis(dimethylsulfinyl) benzene (**96**, **97**) with derivatives (**98**–**101**) and the multi-ring closing reaction of polysulfinyl benzothiophene derivatives (**102**–**104**) catalyzed by the trifluoroacetic acid (TFA)/P_2_O_5_ reagent system (Scheme 18). Due to the high conjugation length, synthesized oligomers (**93**–**95**) showed high emission and quantum yields. Moreover, they were found to be good two-photon absorbers, exhibiting high two-photon absorption coefficients [39]. 

**Scheme 19:**
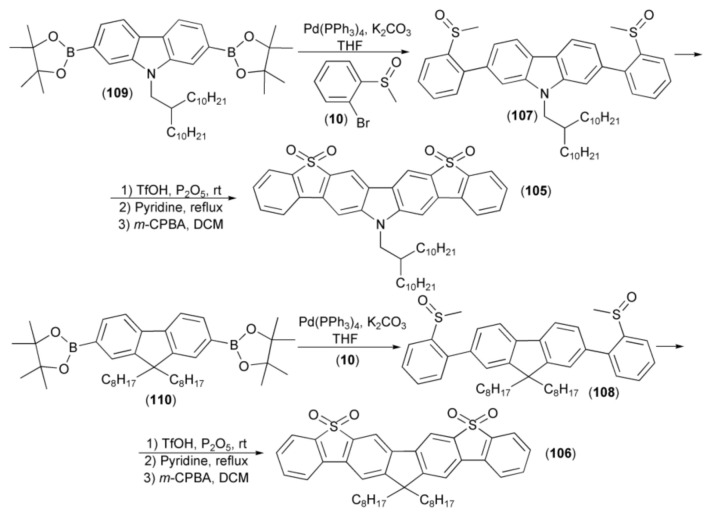
The synthesis of linearly fused polycyclic aromatic compounds (**105**, **106**).

A series of linearly fused polycyclic aromatic compounds (**105**, **106**) were efficiently prepared via the triflic acid (TfOH)-induced intramolecular electrophilic cyclisation reaction of methyl sulfoxides (**107**, **108**). The synthesis of key intermediates (**107**, **108**) was realized through the coupling reaction of the corresponding boronic pinacol esters (**109**, **110**) with 2-bromophenyl methyl sulfoxide (**10**). Final compounds (**105**) and (**106**) showed a deep blue emission with peaks at 426 and 407 nm, respectively. Moreover, the product (**106**) showed a high 77% photoluminescence quantum yield in the tetrahydrofuran solution (Scheme 19) [40].

**Scheme 20:**
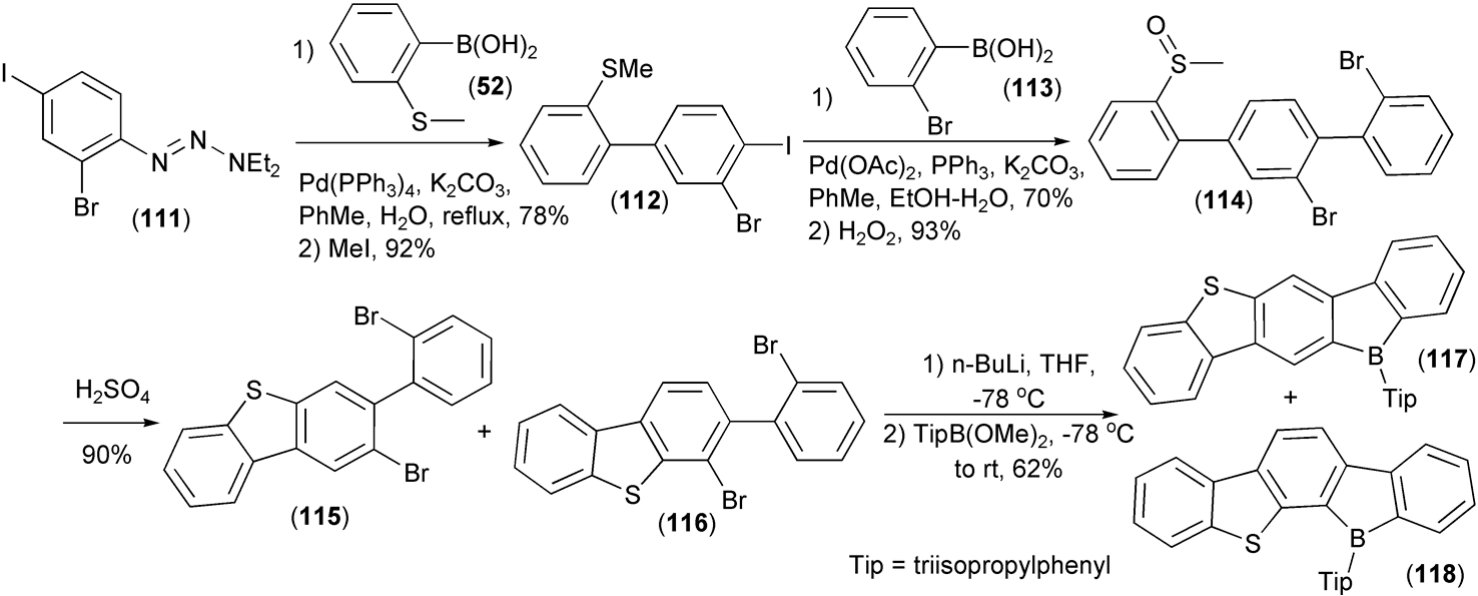
The synthesis of linear heteroacenes (**117**) and (**118**).

The Pd-catalyzed Suzuki–Miyaura coupling reaction of the aryl iodide (**111**) with 2-thioanisylboronic acid (**50**) followed by a subsequent methylation/iodination of the triazeno group in the resulting intermediate, afforded the biphenyl derivative (**112**). The repeated Suzuki–Miyaura coupling of (**112**) with 2-bromophenylboronic acid (**113**) followed by oxidation of the resulting product with H_2_O_2_, delivered the sulfoxide (**114**), which, after the ring-closing condensation with concentrated sulfuric acid, gave a mixture of dibenzothiophene regioisomeric derivatives (**115**) and (**116**). The dilithiation of this mixture with *n*-butyllithium followed by quenching with 2,4,6-triisopropylphenylboronic acid methyl ester (TipB(OMe)_2_) produced a mixture of isomeric linear heteroacenes (**117**) and (**118**). These new ladder-type molecules were highly emissive in both solution and solid state and displayed reversible reduction waves in cyclic voltammograms (Scheme 20) [41].

**Scheme 21:**
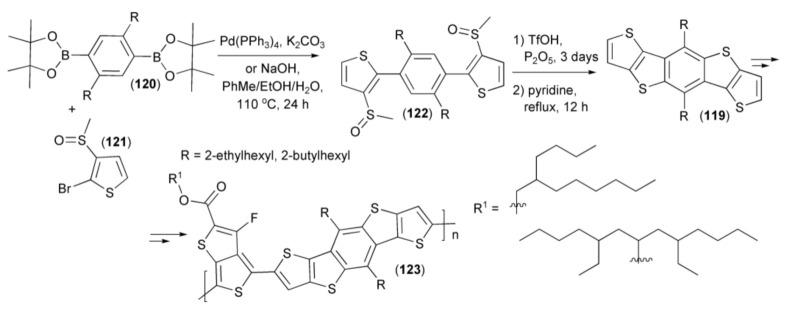
The synthesis monomers (**119**) and their transformation of into push–pull co-polymers (**123**).

Yu and coworkers presented a synthesis of cyclic monomers (**119**) utilizing the reaction sequence involving the Pd-supported coupling of 2,4-benzene diboronic ester (**120**) with 2-bromo-3-methylsulfinylthiophene (**121**) and cyclization of the resulting product (**122**) with triflic acid. Monomers (**119**) were transformed into push–pull co-polymers (**123**) containing branched alkyl chains. The polymer (**123**) showed the highest charge-carrier mobility of 1.69 × 10^−4^ cm^2^/(V s), a suitable balance in charge separation and transport, solubility, and BHJ solar-cell device ((ITO)/poly(3,4-ethylenedioxythiophene): poly(styrenesulfonate) (PEDOT:PSS)/ polymer:PC_71_BM/Ca/Al) efficiency, enhanced up to 7.6% (Scheme 21) [42].

**Scheme 22:**
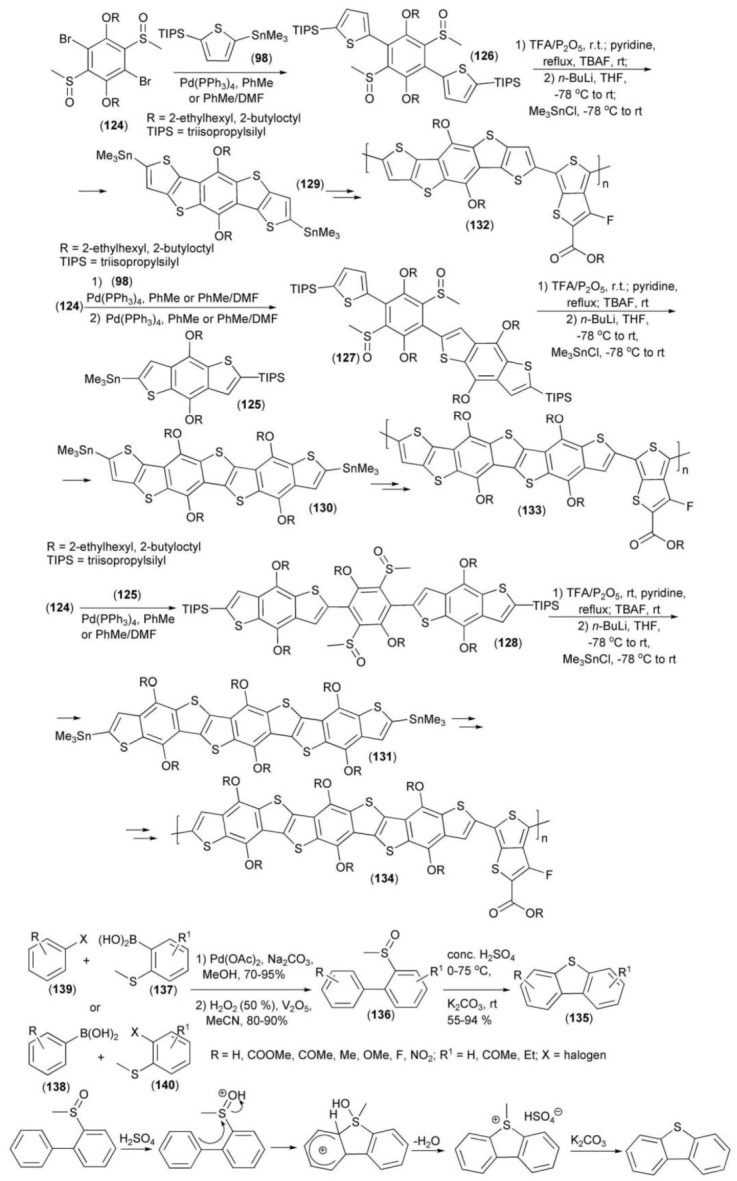
The syntheses of photovoltaic polymers (**132–134**) and their monomers (**129**–**131**).

The Pd-mediated coupling reaction of 2,5-dialkoxy-1,4-dibromo-3,6-bis(methylsulfinyl)benzene (**124**) with derivatives (**98**) or (**125**) in different ratios provided sulfoxide derivatives (**126**–**128**). Cyclization of the latter by a ring-closing reaction followed by lithiation and stannylation generated monomers (**129**–**131**) which can be applied to the synthesis of oligo-heteroacenes with a varied number of fused rings (Scheme 22) [43]. 

The hole mobilities of polymers (**132–134**), measured according to the space charge limited current (SCLC) model, were in the of range 5.02 × 10^−4^–5.56 × 10^−6^ cm^2^/(V s). Photovoltaic properties of these polymers were investigated in a device of the following structure: ITO/PEDOTS:PSS/polymer:PC_71_BM (1:1.5, wt ratio)/Ca/Al. Polymers (**132–134**) revealed a photoconversion efficiency in the range of 0.014–7.6% and light intensity within 0.28-14.5 mA/cm^2^. 

**Scheme 23:**
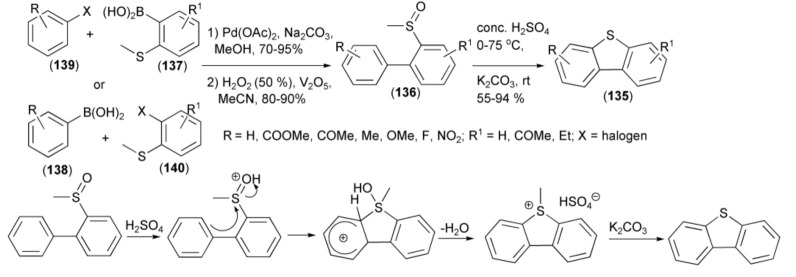
The synthesis of unsymmetrical dibenzothiophenes (**135**).

The synthesis of unsymmetrical dibenzothiophenes (**135**) was achieved by an acid-mediated ring closure of the biphenyl (**136**) having a sulfoxide substituent at the *ortho*-position. The latter was synthesized in the Suzuki coupling reaction of boronic acids (**137**, **138**) with substituted halobenzenes (**139**, **140**) followed by oxidation with hydrogen peroxide (Scheme 23) [44].

**Scheme 24:**
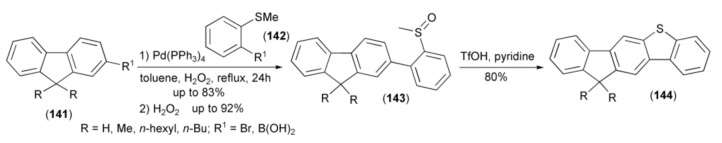
The synthesis of unsymmetrical OLED materials (**144**).

The Suzuki–Miyaura cross-coupling reaction between 9,9-dimethyl-2-fluorene derivative (**141**) and 2-substituted methylthiobenzene (**142**) produced after oxidation of sulfur atom with hydrogen peroxide the methyl sulfoxide derivative (**143**) in 80% yield. The acid-induced condensation of the latter and subsequent demethylation in the presence of water/pyridine afforded unsymmetrical, fused compounds (**144**). The di-*n*-hexyl substituted derivative (**144**) exhibited intense fluorescence in solution with the highest quantum yield of up to 91%. Moreover, the solution-processed, green phosphorescent, organic light-emitting diodes with this derivative as the host material exhibited a maximum brightness of 14,185 cdm^−2^ and a luminescence efficiency of 12 cdA^−1^ (Scheme 24) [19].

**Scheme 25:**
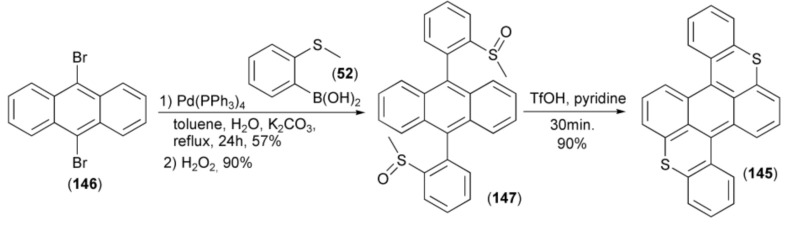
The synthesis of benzobisthioxanthene (**145**).

The synthesis of benzobisthioxanthene (**145**) was realized in the four-step methodology including: the Suzuki cross-coupling reaction of 9,10-dibromoanthracene (**146**) and 2-methylthiobenzene boronic acid (**52**), oxidation of the resulting anthracene derivative with hydrogen peroxide to the precursor 9,10-bis(2-methylsulfinyl-phenyl)anthracene (**147**), cyclization of (**147**) in the presence of triflic acid (TfOH) acid, and finally demethylation in a water–pyridine mixture. The electroluminescent device employing (**145**) as the emitting layer exhibited stable red emission (maximum 40,752 cdm^−2^) and current efficiency up to 4.4 cd A^−1^ (Scheme 25) [45].

## 3. Cyclization of Phosphorus Derivatives

The first example of intermolecular, electrophilic reaction involving a phosphorus precursor with aromatic component under the classical Friedel-Crafts reaction conditions was presented by Maier in 1964 [46]. He found that phosphorus sulfochloride (PSCl_3_) yielded triphenylphosphine sulfide upon reaction with benzene and excess aluminum chloride. Olah and coworkers developed this method for preparation of triphenylphosphine sulfide in 71% yield from benzene and sulfur, phosphorus trichloride, and aluminum chloride [47]. 

**Scheme 26:**
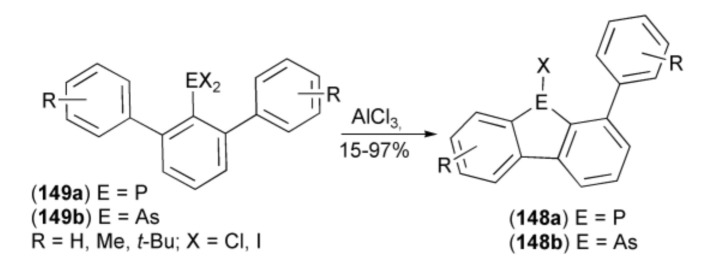
The *phospha*-Friedel-Crafts-Bradsher and related *arsa*-ring-closure reactions.

The AlCl_3_-catalyzed, intramolecular *phospha*- and *arsa*-Friedel-Crafts-Bradsher ring-closure reactions were realized by Wehmschulte and coworkers. They synthesized unsymmetrical 9-chloro-9-phosphafluorenes (dibenzophospholes) (**148a**, E = P) and closely related 9-chloro-9-arsafluorenes (dibenzoarsoles) (**148b**, R = As), starting from *m*-terphenyldichlorophosphines (**149a**) and the corresponding arsines (**149b**) (Scheme 26) [48,49].

**Scheme 27:**
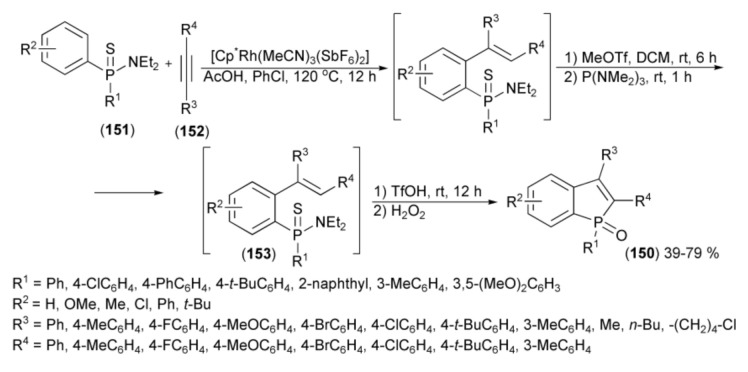
The synthesis of benzo[*b*]phosphole derivatives (**150**).

Miura and coworkers developed a regioselective synthetic sequence leading to benzo[*b*]phosphole derivatives (**150**) via the Rh(III)-catalyzed C-H alkenylation of arylthiophosphinamides (**151**) with alkynes (**152**) followed by the formal *phospha*-Friedel−Crafts-Bradsher cyclization, catalyzed by TfOH and subsequent oxidation of the intermediate (**153**) with hydrogen peroxide (Scheme 27) [50]. 

**Scheme 28:**
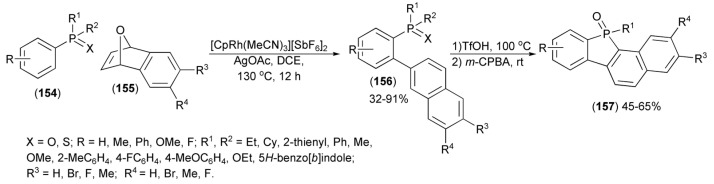
The synthesis of fused dibenzophosphole derivatives (**157**).

Direct coupling of arylphosphine oxides and sulfides (**154**) with the epoxide (**155**) proceeded smoothly under rhodium (III) catalysis and involved hydroarylation followed by dehydrative aromatization to form biarylphosphine derivatives (**156**), which were next transformed to fused dibenzophosphole derivatives (**157**) through the TfOH-mediated intramolecular cyclization in a one-pot manner (Scheme 28) [51].

**Scheme 29:**
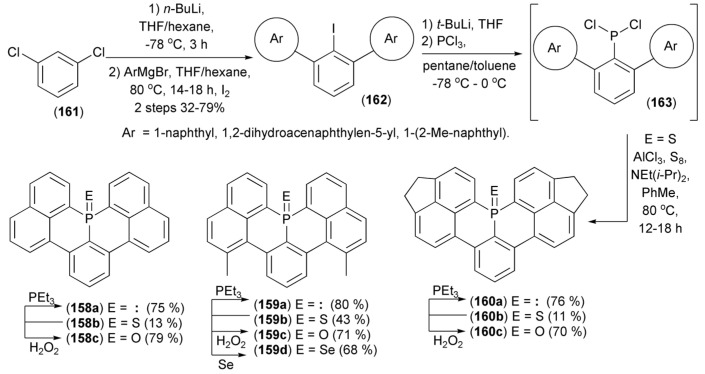
The synthesis of phosphaperylenes (**158**–**160**).

Nakamura and coworkers realized a synthesis of phosphaperylenes (**158–160**) starting from 1,3-dichlorobenzene (**161**). The synthesis involved 1) lithiation at the 2 position in (**161**), 2) subsequent Cl/Ar replacement with Grignard aryl reagents and 3) iodination to deliver teraryls (**162**), and 4) the iodine/lithium replacement in (**162**) followed by phosphinylation with phosphorus trichloride to give the substrate (**163**) for the electrophilic tandem ring-closure in the presence of AlCl_3_/S_8_/NEt(*i*-Pr)_2_ to afford phosphaperylenes (**158**–**160b**, E = S). Other phosphaperylenes (**158–160a**, E = electron pair) were obtained by desulfurization of the latter with triethylphosphine. The corresponding phosphine oxides (**158–160c**, E = O) were synthesized by treatment of the corresponding phosphine sulfides and phosphine selenides (**159d**, E = Se) with H_2_O_2_ (Scheme 29) [52]. 

The obtained phosphaperylenes were thermally stable, and none of the derivatives decomposed below 600 K. Phosphine oxides (**158c**), (**159c**), and (**160c**) exhibited strong blue and cyan fluorescence with maxima (λ_em_) at 418, 415, and 468 nm, respectively. The quantum yield of (**159c**) (Φ_f_ = 0.83) was higher than that of (**158c**) (Φ_f_ = 0.68).

**Scheme 30:**
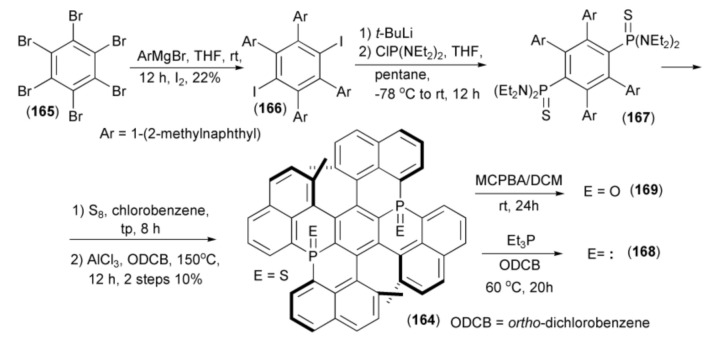
The synthesis of bis(phosphine) helicene derivatives (**164**, **168**, **169**).

The same research group described a synthesis of P-fused double helicene (**164**). In this synthesis, hexabromobenzene (**165**) was treated with Grignard aryl reagents followed by quenching with elemental iodine to give 2,3,5,6-tetraaryl-1,4-diiodobenzene (**166**). The halogen–lithium exchange in the diiodo derivatives (**166**) and subsequent condensation of the resulting aryllithiums with bis(*N,N*-diethylamino) chlorophosphine, gave 2,3,5,6-tetraaryl-1,4-bisphosphine (**167**). Finally, sulfurization and tandem intramolecular *phospha*-Friedel-Crafts-Bradsher reaction of (**167**) with AlCl_3_ yielded the helicene (**164**) (Scheme 30) [53]. 

Desulfurization of (**164**) with triethylphosphine Et_3_P afforded the corresponding bis(phosphine) helicene (**168**) and a selective oxidation of the latter with *m*-chloroperoxybenzoic acid (MCPBA) delivered the corresponding bisphosphine oxide helicene (**169**). Due to the non-planarity of the central ring in (**164**), its aromaticity was reduced but decomposition was not observed even at 600 K under air atmosphere.

**Scheme 31:**
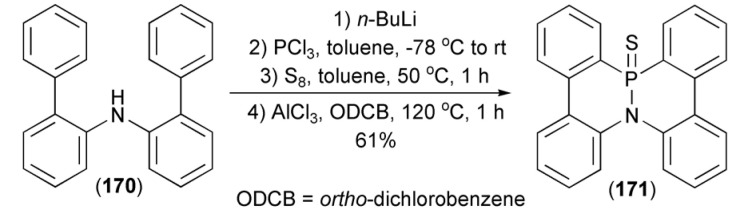
The *phospha*-Friedel-Crafts-Bradsher reaction leading to azaphosphadibenzo[*g*,*p*]chrysene (**171**).

Phosphination of diarylamine (**170**) through the reaction with *n*-butyllithium and PCl_3_ gave diarylaminodichlorophosphine, which after sulfurization with elemental sulfur and the subsequent tandem *phospha*-Friedel-Crafts-Bradsher reaction in the presence AlCl_3_ afforded azaphosphadibenzo[*g*,*p*]chrysene (**171**) in 61% yield (Scheme 31) [5].

**Scheme 32:**
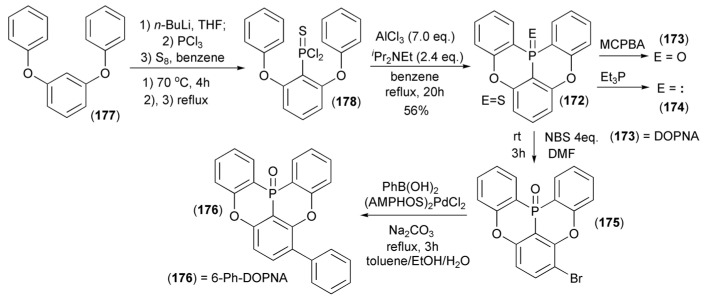
The synthesis of 5,9-dioxa-13b-oxaphosphanaphtho[3,2,1-*de*]anthracene (DOPNA) (**173**) and its derivatives (**174**, **176**).

The Hatakeyama group reported a synthesis of a novel group of cyclic triarylphosphine derivatives (**172–176**) by the direct *ortho*-lithiation of 1,3-diphenoxybenzene (**177**) with *n*-butyllithium and subsequent trapping of the resulting carboanion with PCl_3_ followed by the standard sulfurization of the intermediate phosphine with S_8_ to give arylthiophosphonic acid dichloride (**178**). In the presence of excess of AlCl_3_ and Hünig’s base (NEtiPr_2_), the dichloride (**178**) underwent a tandem *phospha*-Friedel-Crafts-Bradsher reaction at elevated temperature to give 5,9-dioxa-13b-thiophosphanaphtho[3,2,1-*de*]anthracene (**172**) in 56% yield. This polycyclic phosphine sulfide was oxidized with *m*-chloroperbenzoic acid (MCPBA) to afford appropriate phosphine oxide, i.e., 5,9-dioxa-13b-oxaphosphanaphtho[3,2,1-*de*]anthracene (DOPNA) (**173**), or was reduced to the free phosphine (**174**) by Et_3_P (Scheme 32) [54]. 

5,9-dioxa-6-Phenyl-13b-oxaphosph-naphtho[3,2,1-*de*]anthracene (6-Ph-DOPNA) (**176**) was synthesized from (**172**) by electrophilic bromination of the latter using *N*-bromosuccinimide (NBS) in *N*,*N*-dimethylformamide (DMF) followed by Suzuki–Miyaura coupling with phenylboronic acid in the presence of dichlorobis(*p*-dimethylaminophenyldi*tert*-butylphosphine)palladium(II) ((AMPHOS)_2_PdCl_2_) and Na_2_CO_3_. The direct substrate (**172**) was obtained by the reaction sequence involving 2-lithiathion of 1,3-diphenoxybenzene and then the reaction of the resulting lithium derivative with phosphorus trichloride, followed by sulfurization of the dichlorophosphine obtained with elemental sulfur. The DOPNA phosphine derivative (**174**) was synthesized via trans-sulfurization with triethylphosphine (Scheme 32).

**Scheme 33:**
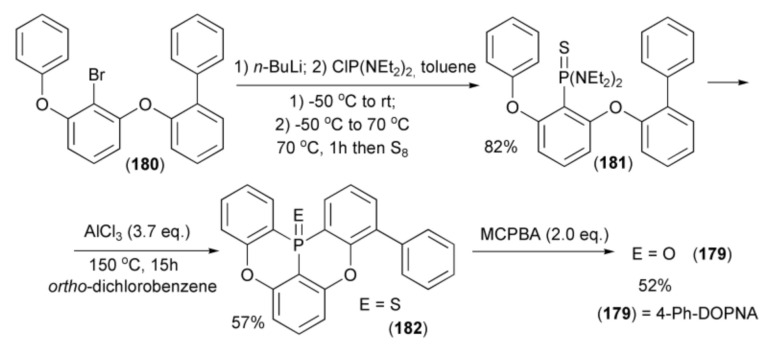
The synthesis of 4-Ph-DOPNA (**179**).

The same research group described the synthesis of 4-phenyl-5,9-dioxa-13b-oxophosphanaphtho-[3,2,1-*de*]anthracene (4-Ph-DOPNA) (**179**) starting from 2-(2-bromo-3-phenoxy-phenoxy)-biphenyl (**180**) by lithium–bromine exchange in (**180**) with *n*-BuLi and by trapping the obtained aryllithium with bis(*N*,*N*-diethylamino)chlorophosphine (ClP(NEt_2_)_2_). Then, sulfurization with S_8_ of the intermediate phosphine amide formed at 70 °C afforded *N*,*N*,*N’*,*N’*-tetraethyl-*P*-arylphosphonothioic diamide (**181**) in 82% yield. The double *phospha*-Friedel-Crafts-Bradsher reaction of (**181**) in the presence of Lewis acid (AlCl_3_) in *ortho*-dichlorobenzene (ODCD) at 150 °C gave the corresponding 4-phenyl-5,9-dioxa-13b-thiophosphanaphtho-[3,2,1-*de*]anthracene (**182**) in 57% yield. Oxidation of the resulting phosphine sulfide by *m*-chloroperbenzoic acid (MCPB) at room temperature afforded (**179**) in 52% yield (Scheme 33). 

Spectroscopic measurements revealed that triplet energy E_T_, ionization potentials I_p_, and optical band gap E_g_ values of these compounds made them suitable PHOLED materials. The E_T_ (estimated from phosphorescence emission maxima) values of DOPNA (2.87 eV), 6-Ph-DOPNA (2.53 eV), and 4-Ph-DOPNA (2.49 eV) were higher than those of Ir(ppy)_3_ (2.45 eV) and BPhen (2.35 eV), which are representative phosphorescence and electron transport materials for phosphorescent organic light-emitting diodes (PHOLEDs). 

To demonstrate the potential of DOPNA derivatives as OLED materials, the authors fabricated phosphorescent organic light-emitting diodes (PHOLED) using these compounds as hole/exciton blocking layer (HBL) components. The addition of 4-Ph-DOPNA and 6-Ph-DOPNA substantially improved the OLED lifetime.

**Scheme 34:**
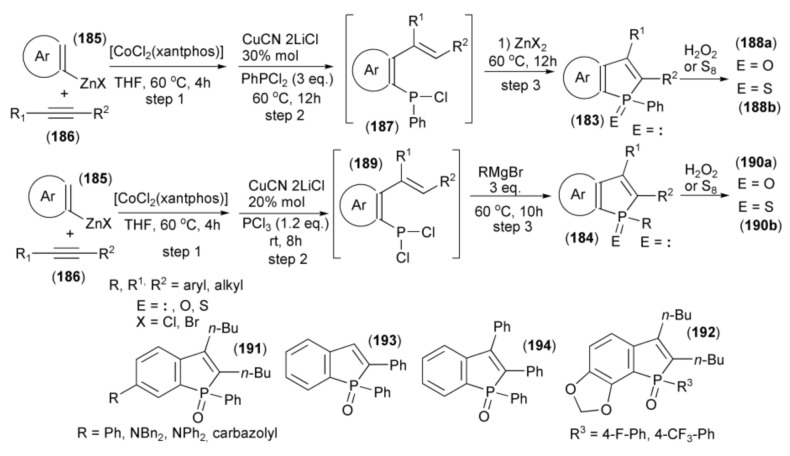
The synthesis of benzo[*b*]phosphole derivatives (**184**, **188a**, **188b**, **190a**, **190b**) starting from arylzinc reagent.

Yoshikai and coworkers showed the synthesis of benzo[*b*]phosphole derivatives (**183**) and (**184**) through a one-pot multicomponent coupling reaction. The starting materials were the arylzinc reagent (**185**) prepared from arylmagnesium bromide and ZnCl_2_ in the presence of TMEDA and alkyne (**186**). The sequential coupling procedure involved a [CoCl_2_(xantphos)]-catalyzed reaction of (**185**) with (**186**) (step 1), the addition of CuCN•2LiCl, and trapping the resulting *ortho*-alkenylarylzinc species with PhPCl_2_ to give aryl phenyl chlorophosphine (**187**) (step 2). The subsequent ring closure in the presence of Lewis acid (ZnX_2_) afforded the final benzo[*b*]phosphole ring system (**183**) (step 3). Oxidation of (**183**) with hydrogen peroxide provided phosphine oxide (**188a**), and sulfurization led to phosphine sulfide (**188b**). Alternatively, in step 2, phosphorus trichloride was used to give the intermediate (**189**) followed by addition of the Grignard reagent RMgBr to substitute one P–Cl bond by aryl and ring closure in one step to afford (**187**) (step 3). Oxidation with H_2_O_2_ or sulfurization with S_8_ gave phosphine oxides or phosphine sulfides (**190a**) or (**190b**), respectively (Scheme 34) [55]. 

Usually, intramolecular *phospha*-Friedel-Crafts-Bradsher cyclization reactions in which P-aryl bonds are formed typically require a strong Lewis acid (for example AlCl_3_) to activate the P–Cl bond; however, this *phospha*-cyclization proceeds in THF under relatively mild conditions in the presence of weak Lewis acid (ZnX_2_). 

Most of the obtained benzophosphole derivatives, especially oxides, were fluorescent in solution. High fluorescence quantum yields (0.57–0.94) were obtained in the case of benzophosphole oxides bearing phenyl, amino, and carbazolyl substituents (**191**). The presence of electron withdrawing substituents on the aryl group at the phosphole phosphorus in the benzophosphole (**191a**) also had a notable effect. Compounds with 4-fluorophenyl and 4-trifluoromethylphenyl (**192**) groups exhibited intense blue emission at 420 and 422 nm, respectively. They also revealed high fluorescence quantum yields of 0.86 and 0.93, respectively. The 2-phenyl and 2,3-diphenyl analogues (**193**) and (**194**) showed λ_abs_ and λ_em_ red-shifts due to extended conjugation at the 2 and 3 positions.

**Scheme 35:**
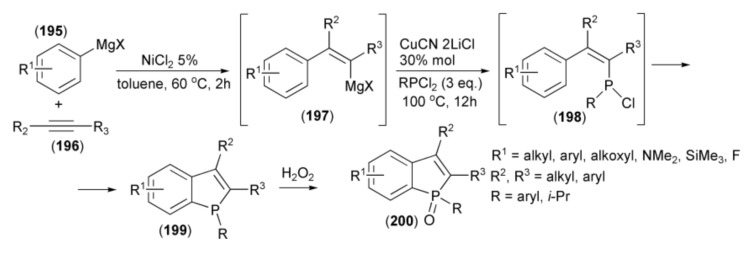
The synthesis of benzo[*b*]phospholes (**200**) starting from arylmagnesium derivatives.

The same research group reported a similar one-pot approach to the synthesis of benzo[*b*]phosphole skeleton utilizing, instead of arylzinc and cobalt salt, a combination of arylmagnesium and nickel salt which allowed inversion of regiochemistry of the *phospha*-cyclization step [56]. Thus, Grignard reagents (**195**) in the presence of NiCl_2_ as a catalyst were added to triple bond of alkynes (**196**) to form the magnesium intermediates (**197**) followed by trapping the resulting *cis*-β-styrylmagnesium species with dichlorophosphine (RPCl_2_) in the presence of CuCN•2LiCl to afford (**198**). A weak Lewis acid (MgX_2_) formed during the reaction, spontaneously catalyzed the *phospha*-Friedel-Crafts-Bradsher cyclization, leading to the benzo[*b*]phosphole ring system (**199**). Oxidation of the latter by hydrogen peroxide afforded the relevant phosphine oxides (**200**) (Scheme 35).

The synthesis of P-chloro-oxaphosphorinane (**201**) usually proceeds via the *phospha*-Friedel-Craft-Bradsher cyclization of the phosphorus dichloride (**202**) in the presence of Lewis acid at very high temperature. The P–Cl bond in (**201**) was next hydrolyzed to *H*-phospinate (**203**). In this approach, the substrate (**205**) was obtained from condensation of 2-hydroxybiphenyl (**204**) with phosphorus trichloride. Later, Pastor et al. and Holmes et al. carried out the same reaction without a solvent using a weaker Lewis acid (ZnCl_2_) at 200 °C [57,58]. 

**Scheme 36:**
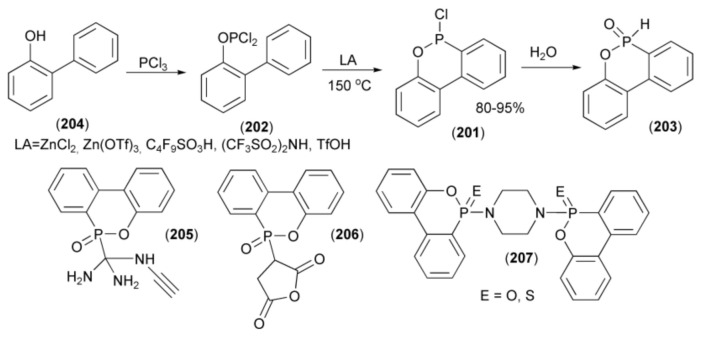
The synthesis of derivatives (**205–207**) as flame retardants and resins hardeners.

In a similar approach, the Ito group described an efficient synthesis of *6H*-dibenz[*c,e*][1,2]oxaphosphorin-6-chloride (**201**) using other strong acids, such as Zn(OTf)_3_, C_4_F_9_SO_3_H, (CF_3_SO_2_)_2_NH, or TfOH. Moreover, the reaction conditions were milder (120–150 °C) than in previous syntheses (Scheme 36) [59]. 

P-Functionalized derivatives (**205–207**) revealed a possibility of interesting applications [60]. For example, the derivative (**205**) was investigated as flame retardant and hardener for epoxy resin based on bisphenol A, bisphenol A-novolac, and *o*-cresol novolac. The compounds (**206**) and (**207**) were used as flame retardants additive for poly(butylene terephthalate) and epoxy resin (DEN 438/DICY/Funoron), respectively. These derivatives produced phosphoric and polyphosphoric acids during thermal decomposition process, which acted as a heat-resistance barrier with a flame inhibition characteristic in gas phase.

**Scheme 37:**
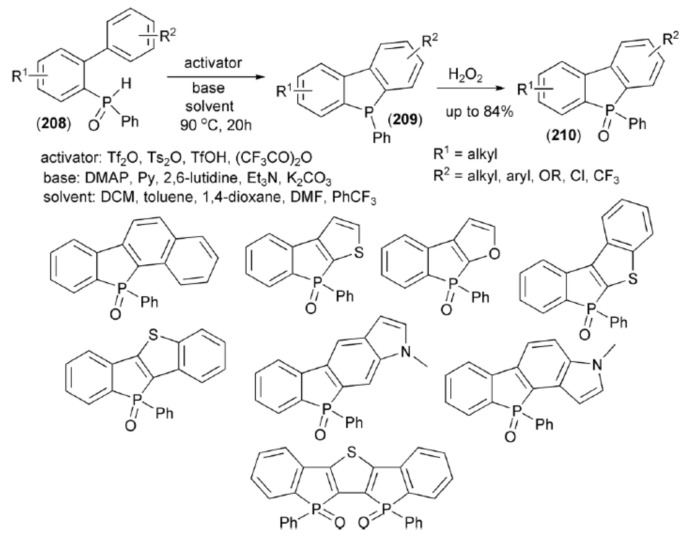
The Tf_2_O-mediated synthesis of dibenzophospholes (**210**).

Hirano and Miura presented an application of the Tf_2_O-mediated *phosp*ha-Friedel-Crafts-Bradsher cyclization in the synthesis of dibenzophospholes. Secondary biarylphosphine oxides (**208**) underwent tautomerization to the corresponding hydroxyphosphines. Their OH group was activated with Tf_2_O to deliver an electrophilic phosphorus center which could be readily trapped with the proximal aromatic ring to give (**209**). Oxidation of (**209**) with H_2_O_2_ afforded a family of dibenzophosphole oxide (**210**) (Scheme 37) [61]. 

**Scheme 38:**
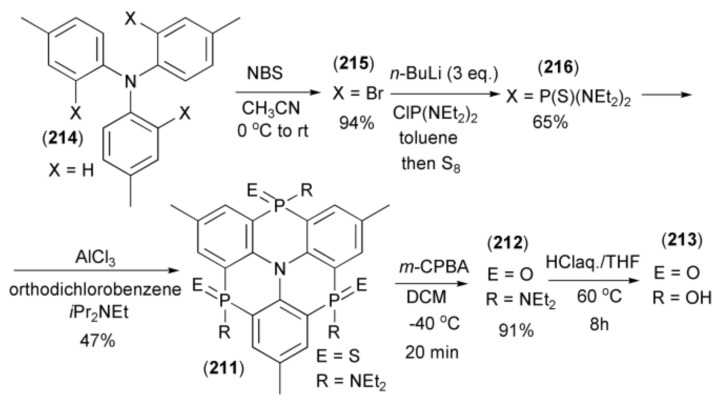
The synthesis of triphosphaazatriangulenes (**211**–**213**) through the triple *phospha*-Friedel-Crafts-Bradsher cyclization.

The Hatakeyama group reported synthesis of triphosphaazatriangulenes (**211–213**) through triple *phospha*-Friedel-Crafts-Bradsher cyclization [62]. The triangulene (**213**) was synthesized in five steps starting from tri-*p*-tolylamine (**214**), which was brominated with *N*-bromosuccinimide (NBS) to give the corresponding tribromide (**215**). Triaryllithium, generated from (**215**) by *n*-butyllithium, was trapped with excess of bis(*N*,*N*-diethylamino)chlorophosphine (ClP(NEt_2_)_2_) and submitted to sulfurization with elemental sulfur to afford the intermediate tris(thioamide) (**216**). The triple cyclization of (**216**) with Hünig’s base in the presence of AlCl_3_, at elevated temperature, afforded triphosphaazatriangulene (**211**) (Scheme 38). Oxidation of the latter with MCPBA and hydrolysis by HCl_aq._ in THF, gave the corresponding triangulenes (**212**) and (**213**). 

The presence of a phosphinic acid group (P(O)(OH)) in the triangulene (**213**) indicated that its complex with copper (**213**)•Cu_2_(OH)_4_) could be a highly proton-conducting material. The triangulene was subjected to AC impedance spectroscopy under conditions of controlled humidity. The proton conductivity was found to be 5.9 × 10^−8^ S cm^−1^ at 25 °C and 55% relative humidity (RH). Nyquist plots, under a variety of humidity conditions (from 55% to 95% RH) at 25 °C, showed that conductivity increased with increasing RH.

## 4. Conclusions

Introduction of heteroatoms into cyclic, π-conjugated molecules usually delivers new materials with enhanced properties compared to carbon analogs. This was the case with five- and six-membered heteroacenes containing ring phosphorus and sulfur atoms.

These two heteroatoms have been introduced into carbon backbones by harnessing the classical Friedel-Crafts and Bradsher reactions in new heteroatomic versions (*thia*- and *phospha*-). The review demonstrates the progress made in both types of reactions, leading to construction of a variety of benzo- and thieno-fused, carbo- and heterocyclic frameworks which gained applications, mainly as semiconducting and light emitting materials in organic diodes, transistors, and photovoltaic devices. Extensive literature and a variety of examples show that this type of reaction has become an important synthetic tool in modern organic chemistry for introduction of ring phosphorus and sulfur into molecular structures. We believe that this review will serve as a useful reference for chemists interested in developing libraries of new *thia*- and *phospha*-derivatives and in extending their applications over emerging areas. This review should also stimulate the development of the chemistry of arsenic and selenium, the two heavier neighbors of sulfur and phosphorus in the periodic table, based on electrophilic aromatic substitution reaction.
